# Technical evaluation of the cone‐beam computed tomography imaging performance of a novel, mobile, gantry‐based X‐ray system for brachytherapy

**DOI:** 10.1002/acm2.13501

**Published:** 2021-12-14

**Authors:** Andre Karius, Marek Karolczak, Vratislav Strnad, Christoph Bert

**Affiliations:** ^1^ Department of Radiation Oncology Universitätsklinikum Erlangen Friedrich‐Alexander Universität Erlangen‐Nürnberg Universitätsstraße 27 Erlangen Germany; ^2^ Comprehensive Cancer Center Erlangen‐EMN (CCC ER‐EMN) Erlangen Germany; ^3^ Institute of Medical Physics Friedrich‐Alexander‐University Erlangen‐Nuremberg Henkestraße 91 Erlangen Germany

**Keywords:** cone‐beam computed tomography (CBCT), image‐guided adaptive brachytherapy, image quality, imaging performance analysis, mobile X‐ray system

## Abstract

**Purpose:**

A novel, mobile cone‐beam computed tomography (CBCT) system for image‐guided adaptive brachytherapy was recently deployed at our hospital as worldwide first site. Prior to the device's clinical operation, a profound characterization of its imaging performance was conducted. This was essential to optimize both the imaging workflow and image quality for achieving the best possible clinical outcomes. We present the results of our investigations.

**Methods:**

The novel CBCT‐system features a ring gantry with 121 cm clearance as well as a 43.2 × 43.2 cm^2^ flat‐panel detector, and is controlled via a tablet‐personal computer (PC). For evaluating its imaging performance, the geometric reproducibility as well as imaging fidelity, computed tomography (CT)‐number accuracy, uniformity, contrast‐noise‐ratio (CNR), noise characteristics, and spatial resolution as fundamental image quality parameters were assessed. As dose metric the weighted cone‐beam dose index (CBDI_w_) was measured. Image quality was evaluated using standard quality assurance (QA) as well as anthropomorphic upper torso and breast phantoms. Both in‐house and manufacturer protocols for abdomen, pelvis, and breast imaging were examined.

**Results:**

Using the in‐house protocols, the QA phantom scans showed altogether a high image quality, with high CT‐number accuracy (*R* ^2 ^> 0.97) and uniformity (<12 Hounsfield Unit (HU) cupping), reasonable noise and imaging fidelity, and good CNR at bone–tissue transitions of up to 28:1. Spatial resolution was strongly limited by geometric instabilities of the device. The breast phantom scans fulfilled clinical requirements, whereas the abdomen and pelvis scans showed severe artifacts, particularly at air/bone–tissue transitions.

**Conclusion:**

With the novel CBCT‐system, achieving a high image quality appears possible in principle. However, adaptations of the standard protocols, performance enhancements in image reconstruction referring to artifact reductions, as well as the extinction of geometric instabilities are imperative.

## 1 INTRODUCTION

Since the beginning of the 21st century, cone‐beam computed tomography (CBCT) has progressively established as important part of the imaging workflows in several medical fields,^1^, ^2^, ^3^, ^4^, ^5^, ^6^, ^7^, ^8^, ^9^ such as dentistry,^1^ orthopedics,^2^ image‐guided radiotherapy,^3^, ^4^ and brachytherapy.^5^ Due to their often mobile configuration,^6^, ^7^, ^8^ corresponding CBCT‐scanners are particularly suited and reveal high potential for both intraoperative^6^, ^7^ and interventional^8^ applications.

Modern CBCT‐devices are equipped with a digital flat‐panel detector (FPD) and cone‐beam emitting X‐ray source, both mounted to the system's gantry. With this setup, planar images of the entire anatomical region of interest (ROI) are acquired from several hundred to thousand different projection angles, forming the basis for the reconstruction of a 3D field of view (FOV).^9^ The resulting CBCT‐images are in general characterized by high isotropic spatial resolution in the submillimeter range^9^, ^10^ with appropriate geometric accuracy.^10^, ^11^ Drawbacks, especially compared to conventional multi‐slice computed tomography (CT), are mainly increased image noise, reduced image uniformity, as well as inferior low‐contrast differentiability.^9^, ^12^ Respective image quality improvements are subject of ongoing research.^13^, ^14^, ^15^ For instance, Sheth et al.^13^ demonstrated improved low‐dose performance with respect to noise, resolution, and detective quantum efficiency using complementary metal‐oxide‐semiconductor (CMOS) detectors instead of common SI:H‐based FPDs. Jin et al.^14^ substantially reduced the amount of image artifacts and noise by combining a direct reduction and respective software‐sided correction of scatter radiation. Kida et al.^15^ increased uniformity and signal‐noise‐ratio using a deep convolutional neural network.

For image‐guided adaptive brachytherapy, the novel, mobile CBCT‐system ImagingRing m (IRm; medPhoton, Salzburg, Austria) was recently deployed at our hospital as worldwide first site. The IRm features a 43.2 × 43.2 cm^2^ FPD, 121 cm gantry clearance, battery‐powered maneuverability, and full remote control via a tablet‐personal computer (PC). This ensures high flexibility and mobility, facilitates both interventional and intraoperative imaging, and distinguishes the device from previous X‐ray systems.

Prior to its clinical operation, a profound characterization of the IRm's imaging performance was essential. This procedure allows, by assessing objective image quality and dose parameters, to draw conclusions about required adaptions of existing imaging workflows and/or the device's performance to underlying clinical requirements. Comparable characterizations of previous X‐ray systems^6^, ^7^, ^13^, ^16^ included, for instance, the assessment of noise characteristics, contrast behavior, spatial resolution, and uniformity as fundamental image quality parameters.

The scope of the present work is the technical assessment of the CBCT imaging performance of the IRm. For this purpose, both scan protocols provided by the manufacturer as well as in‐house protocols adapted to clinical requirements were used. In addition to a standard quality assurance (QA) phantom for determining physical imaging parameters, anthropomorphic phantoms were examined for evaluating the subjective quality of scans of different anatomical ROIs.

## 2 MATERIALS AND METHODS

### 2.1 ImagingRing m

**FIGURE 1:**
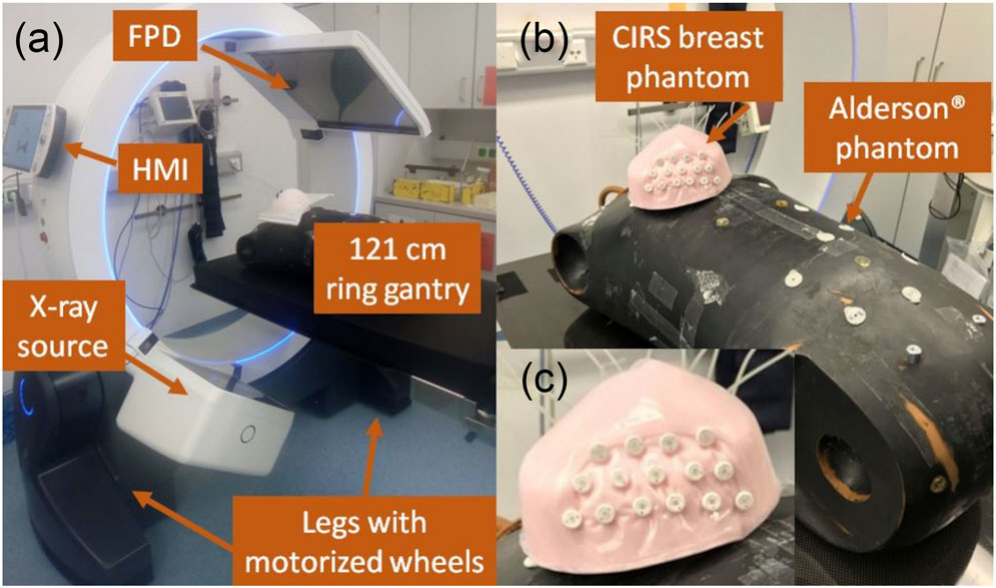
Shown is the technical setup of the IRm (a), which is constructed from an X‐ray source and flat‐panel detector (FPD) mounted on a gantry with 121 cm clearance and controlled via the portable Human Machine Interface (HMI). Motorized wheels integrated to the system's legs ensure high mobility. Particularly, an Alderson® upper torso as well as a CIRS breast phantom (b), in which plastic catheters were implanted, were used for imaging assessment of the IRm. (c) A zoom image of the breast phantom is shown

The IRm is a novel X‐ray system featuring CBCT, radiography, and fluoroscopy as imaging modalities. It is built upon an aluminum ring gantry with 121 cm clearance, on which an X‐ray source and FPD are mounted independently movable (Figure 1). Both source and detector can rotate about more than 650° around the gantry's rotational axis driven by cable pulls (thus being able to perform, depending on start/end‐positions of source and detector, 2–3 scans without having to change the rotation direction) and be arranged at any angular position with an accuracy of 0.1°. The individual positioning of source and detector enables non‐isocentric as well as stitched volumetric and planar imaging. Technical specifications of gantry, source, and detector are given in Table 1.

**TABLE 1:** Technical characteristics of the IRm's gantry, source, and detector

Geometrical construction	
Physical dimensions	182 × 87 × 190 cm^3^
Gantry bore	121 cm clearance
Source axis distance	74.3 cm
Source detector distance	126 cm
Orbital gantry range	About 650°
Source characteristics	
Generator	HF1 GMX‐350/S2 (IMD)
Anode	RTM 780H 0.3/0.6 (IAE)
Focal spot	0.3 mm (max. power: 6 kW)
	0.6 mm (max. power: 25 kW)
Tube current	0.01–120 mA
Tube voltage	60–120 kV (in steps of 10 kV)
Pulse length	2–35 ms
Pulse rate	Up to 30 Hz
Prefiltering	Air; 1.5 mm Al; pelvis Cu‐Bowtie; 0.2, 0.5, and 1.5 mm Cu
IRm's inherent filtration	4.4 mm Al equivalent (at 75 kV)
Detector characteristics	
Model	XRD4343RF (Varex Imaging)
Active area	43.2 × 43.2 cm^2^
	2880 × 2880 pixels
Pixel pitch	150 μm (1 × 1 binning)
Binning modes	1 × 1, 2 × 2, 3 × 3 binning
Read‐out frame rate	12 fps
Scintillator	Direct deposit CsI:TI
Detector array	Single substrate amorphous silicon active TFT diode array
	

For generating an X‐ray cone‐beam, the IRm is equipped with a HF1 GMX‐350/S2 (IMD, Grassobio BG, Italy) generator with integrated anode of type RTM 780H 0.3/0.6 (IAE, Cormano Milano, Italy). This setup enables imaging with two focal spots of 0.3 mm (maximum tube‐current: 30 mA, maximum power: 6 kW) and 0.6 mm (120 mA, 25 kW) size. Examinations can be performed at tube voltages of 60–120 kV (in 10 kV steps) with both continuous or pulsed (pulse lengths: 2–35 ms) tube output.

The IRm provides a fixed inherent minimum filtration equivalent to 4.4 mm aluminum (at 75 kV). For additional spectral hardening, an independently adjustable filter wheel and carriage are integrated within the beam path of the device. The filter wheel contains air, 1.5 mm Al, 0.2 mm Cu, and 0.5 mm Cu as prefilters. The filter carriage comprises air, 1.5 mm Cu, as well as a Bowtie filter specified for the pelvic region. Moreover, within a so‐called volume‐definition workflow (VDW), four independently movable jaws serve to dynamically collimate the X‐rays towards the actual anatomical ROI only. Within this VDW, an anterior–posterior (AP) and lateral topogram is acquired, based on which the 3D FOV can be specified prior to a CBCT acquisition. The jaws enable for each projection a maximum planar isocentric FOV of 25.4 × 25.4 cm^2^.

For image acquisition, the IRm features a XRD4343RF (Varex Imaging, Salt Lake City, UT, USA) FPD, which consists of a direct deposit CsI:Tl scintillator that is connected via fiber optic plate to a single substrate amorphous silicon active thin‐field transistor diode array. The detector has an active area of 43.2 × 43.2 cm^2^ and can be operated in 1 × 1 (pixel pitch: 150 μm, 2880 × 2880 pixels), 2 × 2, and 3 × 3 binning modes. It is currently read out with 12 Hz frame rate.

The operation of the IRm is performed via WiFi‐based remote control by means of a portable so‐called Human Machine Interface (HMI, Figure 1), which is established on a Windows tablet‐PC equipped with additional joysticks and buttons. As visualized in the supplementary materials, motorized wheels integrated into the system's legs (Figure 1) as well as gearboxes enable longitudinal and lateral translation movements, free rotations on the floor, and up to ±30° gantry tilt. All maneuvers can particularly be carried out in a battery mode for up to 30 min. Manually moving the device without the HMI is currently not possible in standard operation. However, for emergency situations such as battery failure, emergency wheels can be extended from the legs via hand cranks, which allow the IRm to be moved manually in longitudinal direction.

Four system cameras, two integrated in the gantry and two in the detector, allow patient monitoring during ongoing examinations. The cameras are particularly suited also for real‐time tracking of medical instruments, for example, for image‐guided surgery. In addition, built‐in lasers serve to align the system for scans and to visualize the FOV of examinations directly on the patient's skin. This facilitates both the exact positioning of the device and the imaging workflow.

### 2.2 Scan protocols

The scope of the present work is the assessment of the CBCT imaging performance of the IRm. Both standard scan protocols provided by the manufacturer and protocols developed in‐house were used. In particular, in‐house protocols for breast and pelvis imaging were defined based on our CBCT experience, as these anatomical regions represent our main clinical focus for image‐guided adaptive brachytherapy and the corresponding manufacturer's protocols did not fulfill our clinical requirements. The scan parameters of the protocols investigated in this work are listed in Table 2. In the following, for brevity, all protocols are referred to by the abbreviations listed in Table 2. Note that the IRm does not feature any automatic exposure control, which is why image acquisitions generally have to be explicitly adapted to different patient sizes/characteristics: for the in‐house protocols, all parameters were determined based on experience; for the manufacturer protocols, the parameters resulted automatically from selecting different dose levels (low, medium, high) within the IRm's control software.

**TABLE 2:** Listed are the main scan parameters (scan time, tube voltage, current‐time‐product per frame prior to prefiltering, number of frames, prefiltering, reconstruction kernel with frequency cutoff as fraction of the Nyquist frequency in brackets, velocity modulation (VM), and focal spot (FS) size) of all examined protocols

	Parameters							
Protocol	Time (s)	Voltage (kV)	Tube output (mAs)	Frames	Prefilter	Kernel	VM	FS (mm)
Custom protocols								
Pelvis								
‐ Low dose (PL)	20	120	0.60	240	0.2 mm Cu	CO (0.9)	–	0.3
‐ Standard (PS)	25	120	1.16	300	0.5 mm Cu	SL (0.8)	2.5:1	0.6
‐ Obese (PO)	25	120	1.50	300	0.5 mm Cu	SL (0.8)	2.5:1	0.6
‐ High‐quality 360° (PH)	50	120	1.80	600	1.5 mm Cu	SL (0.8)	2.5:1	0.6
Breast								
‐ Standard (BS)	17	110	0.90	204	0.5 mm Cu	SL (0.8)	–	0.3
‐ Obese (BO)	17	120	0.96	204	0.5 mm Cu	CO (0.9)	–	0.6
‐ High‐quality (BH)	30	120	1.50	360	1.5 mm Cu	SL (0.8)	–	0.6
Standard presets								
Abdomen								
‐ Low dose (AL)	26	100	0.09	312	Air	RL	2.5:1	0.3
‐ Standard (AS)	26	100	0.56	312	Air	RL	2.5:1	0.6
‐ Obese (AO)	26	100	2.10	312	Air	RL	2.5:1	0.6

*Note*: Both in‐house protocols for pelvis and breast as well as manufacturer protocols for abdomen imaging were investigated. A Cosine (CO), Shepp–Logan (SL), and RamLak (RL) kernel were used.

The IRm can perform both short scan (orbital range: 180° + beam divergence) and full scan (360°) trajectories. In this work, we acquired all scans using the VDW and short scans with source right orbit. These allow lower acquisition times (at least 12.5 s) than full scans (at least 22.5 s) and, at fixed scan time, improved angular sampling. Only for the pelvis protocol PH, with the intention of image quality enhancements by avoiding potential short scan artifacts, a full scan trajectory with extended acquisition time was resorted to. For both breast and pelvis, the so‐called high‐quality protocols BH and PH referred to the use of the strongest prefiltering (1.5 mm Cu). Due to the high absorption of this prefilter, BH and PH featured a comparatively increased current‐time‐product and a significantly extended acquisition time, which also improved angular sampling. With the exception of PL, all pelvis protocols exhibited 2.5:1 velocity modulation (thus reducing the travel speed of source/detector in the lateral angular range by a factor of 2.5 compared to the AP range), to increase the overall dose rate in the lateral regime.

The manufacturer's protocols for the abdomen as third anatomical ROI were adopted directly from the IRm's control software without any adaptions. These were characterized in particular by using air prefiltering and 2.5:1 velocity modulation.

Image reconstruction is based on a variation of the Feldkamp–David–Kress algorithm,^17^, ^18^ starts already during running examinations in a so‐called real‐time reconstruction, and typically finishes within less than 50 s after scan completion. For the in‐house protocols, a Shepp–Logan reconstruction kernel with cutoff 0.8 (fraction of the Nyquist frequency) was used in general. Only for the entity‐specific protocols yielding the highest noise magnitudes in patient examinations (PL and BO), a Cosine kernel with cutoff 0.9 was utilized to reduce noise by slightly enhanced image smoothing. The manufacturer's abdomen protocols featured a RamLak kernel with cutoff 1.0. It has to be mentioned that the IRm's control software currently does not allow repeated (re‐)reconstructions of acquired scans, and thus a retrospective adjustment of image parameters such as kernel or slice thickness is not directly possible. In general, the IRm is able to perform reconstructions using a RamLak, Shepp–Logan, Cosine, Hamming, or Hann kernel each with manually adjustable cutoff frequency.

All scans were acquired using the 2 × 2 detector binning. Within the reconstruction process, the scans were reconstructed initially with a voxel size of 0.2 × 0.2 × 0.5 mm^3^ and the re‐binned to the actual set voxel size of 0.4 × 0.4 × 1 mm^3^ output to the user. The FOV had dimensions of about 20 × 20 × 20 cm^3^.

### 2.3 Geometric reproducibility

The geometric accuracy of CBCT‐scanners is particularly subject to the influence of mechanical/gravitational sag and flex of scanner components such as source and detector. These intrinsic properties might cause deviations between the center of actual imaging and reconstruction, resulting in degradations of image contrast as well as misalignment artifacts.^19^, ^20^, ^21^ Consequently, there is a need to account for geometric inaccuracies in the scan trajectory and thus to calibrate the scanner geometry accordingly.

For geometric calibration of the IRm, the flexmap approach^21^, ^22^ is pursued by using a cylindric nine‐degrees‐of‐freedom ball bearing calibration phantom^21^ that is supplied together with the IRm. The phantom is placed on the patient table after consultation with the manufacturer, fixed to a stable holder to prevent rolling. By means of this phantom, three (one for each spatial dimension *x*, *y*, *z*) translation corrections tC*_x_*, tC*_y_*, tC*_z_* and tS*_x_*, tS*_y_*, tS*_z_* of the detector midpoint C and focal spot S, respectively, as well as three Euler rotation angular corrections *r_x_*, *r_y_*, *r_z_* of the detector's row‐/column‐vectors are determined as function of the gantry angle. This is done as described in detail by Keuschnigg et al.^21^ and within the IRm's default calibration method following the manufacturer's standard operating procedure. As defined by this group, the *x*‐component refers to the gantry's rotation direction, *y* to the direction along the rotational axis, and *z* to the radial direction within the tomographic plane. The calibration procedure involves a gantry angular range of 520° with 53 projection views in 10° steps. Clockwise and counter‐clockwise gantry rotations are considered separately. The objective of the calibration is to ensure geometric accuracy in the reconstruction by applying the determined corrections angular‐dependent to each projection of a scan.

The geometric reproducibility of the IRm was validated by performing the described procedure eight times in direct succession and analyzing the angular‐dependent variations of the obtained corrections. The consistency of the individual measurements was evaluated by calculating both the mean offsets and the standard deviations of the results. The impact of the gantry's rotation direction was assessed by calculating the differences of the corrections obtained for clockwise and counter‐clockwise gantry rotation for each calibration.

### 2.4 Physical imaging parameters

For assessing the CBCT image quality, the CatPhan® 504 (CatPhan; The Phantom Laboratory, Salem, NY, USA) was used. This is a modular phantom consisting of four individual segments, a detailed description of which is provided by the manufacturer.^23^ The phantom was placed isocentrically within the scanner on a carbon fiber table, unless otherwise mentioned, and served for evaluating imaging fidelity, CT‐number accuracy, contrast‐noise‐ratio (CNR), noise characteristics, uniformity, and spatial resolution. Within the frame of this work, all considered image metrics were measured eight times each.

#### 2.4.1 Imaging fidelity

**FIGURE 2:**
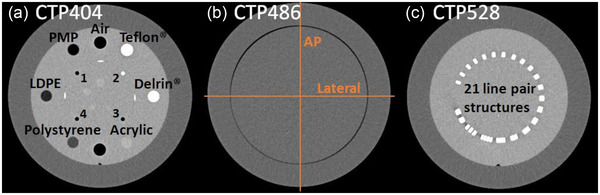
The CatPhan modules CTP404 (a), CTP486 (b), and CTP528 (c) used for physical image analysis. For uniformity assessment, computed tomography (CT)‐number profiles were drawn within the CTP486 along the indexed directions (b). The four rods used for the assessment of imaging fidelity are labeled with the numbers 1–4. Images from our archive, acquired with the conventional CT‐system SOMATOM go.Open Pro (Siemens Healthineers, Erlangen, Germany)

In addition to the geometric reproducibility (Section 2.3), the imaging fidelity is of high importance for evaluating a scanner's geometric accuracy. In other words, it must be ensured that the real lengths of examined objects are accurately reflected on the scans. To quantify the IRm's imaging fidelity, we considered the four rods of the CatPhan module CTP404, which are arranged as square with side length 50 mm (Figure 2a). On the module's central axial slice, the Euclidean distances between the centers of the individual rods, that were determined via threshold‐based detections, were measured pairwise. For each scan protocol, the absolute difference between the measured lengths and the real rod distances was averaged over all rod pairings (i.e., both the side and diagonal lengths of the square were considered) and all scans. This provided a quantitative measure of the geometric accuracy of the device. The standard deviation of the obtained differences corresponded to the associated geometric imaging uncertainty.

#### 2.4.2 CT‐number accuracy

CT‐number accuracy was assessed by considering the seven inserts of the CatPhan module CTP404 (Figure 2a), embedded in a uniform background material.^23^ On the central axial slice of this module, a circular ROI was centered within each insert and the respective mean CT‐number was measured.

#### 2.4.3 Contrast‐noise‐ratio

Based on the central axial slice of the CTP404 module, the CNR of the low density polyethylene (LDPE, similar CT‐number to fat), polystyrene (soft tissue), and Delrin® (bone) insert in relation to the module background was calculated. These inserts were chosen, since they represent a broad human‐body like CT‐number spectrum. As reported in detail previously,^6^ the CNR was computed based on the CT‐number mean ROI_insert_ and standard deviation *σ*_insert_ of a circular ROI centered within each insert as well as ROI_bkg_ and *σ*_bkg_ of an adjacent background ROI:

(1) $$\mathrm{CN}R_{\mathrm{insert}}=\frac{\left|\mathrm{RO}I_{\mathrm{bkg}}-\mathrm{RO}I_{\mathrm{insert}}\right|}{\sqrt{\frac{1}{2}\cdot \left(\sigma_{\mathrm{bkg}}^{2}+\sigma_{\mathrm{insert}}^{2}\right)}}.$$

#### 2.4.4 Noise characteristics

Furthermore, we characterized both magnitude and spectral composition of observed image noise. For this purpose, the 2D noise power spectrum (NPS) as function of the spatial frequencies *f_x_*_,_ *_y_* (for both in‐plane image directions *x* and *y*) was calculated for each scan. Out of two central slices of the homogeneous CatPhan module CTP486 (Figure 2b), a difference image was computed. The two slices were spaced four times the slice thickness to avoid cross‐correlation effects. An ensemble of $N_{\mathrm{ROI}}$ ROIs ($N_{\mathrm{ROI}}$ > 200) of size 5 × 5 cm^2^ was uniformly distributed within the CTP486 on this difference image, and the 2D NPS was obtained as proposed by Steiding et al.^10^ (for more in‐depth description of the calculation, please refer to this original article):

(2) $$\mathrm{NPS}\left(f_{x},\;f_{y}\right)=\frac{1}{N_{\mathrm{ROI}}}\cdot \frac{\Delta x\Delta y}{N_{x}N_{y}}\cdot \sum_{i\;=\;1}^{N_{\mathrm{ROI}}}\frac{|\mathrm{DFT}\left\{\mathrm{RO}I_{i}-CT_{i}\right.\}{|}^{2}}{2}.$$

As described by this group,^10^ Δ*x*, Δ*y* correspond to the in‐plane pixel sizes and *N_x_*, *N_y_* correspond to the numbers of pixels of a ROI in *x*‐ and *y*‐directions, respectively. The discrete Fourier transform (DFT) of the ROIs $\mathrm{RO}I_{i}$, that were offset‐corrected with their respective mean CT‐number $CT_{i}$, allows the examination of the spectral composition of image noise in the frequency domain. The factor 1/2 accounts for the artificial noise increase induced by the difference image approach.^10^

For visualization, the 1D NPS NPS(*f_r_*) (with $f_{r}^{2}=f_{x}^{2}+f_{y}^{2}$) was computed by radial averaging of the 2D NPS. Discrete integration of the 1D NPS yielded the magnitude *σ*_NPS_ of the image noise.

#### 2.4.5 Spatial resolution

Spatial resolution of scans was assessed by considering a non‐resolvable line pair structure, that was slightly rotated within the axial plane, of the CatPhan module CTP528 (Figure 2c). Several line profiles were drawn across this structure, and by subsequent appropriate superposition an oversampled edge‐spread function was created.^24^ The latter was fitted with a Fermi edge function and derived to obtain the line spread function LSF(*x*), which was resampled to one‐tenth of the pixel size of the scan. By DFT, the modulation transfer function MTF(*f_x_*) dependent on the spatial frequency *f_x_* was calculated:

(3) $$\mathrm{MTF}\left(f_{x}\right)=\left|\;\mathrm{DFT}\left\{\;\mathrm{LSF}(x)\right\}\;\right|.$$

The MTF was normalized to MTF(0).

#### 2.4.6 Uniformity

For evaluating image uniformity, 10 image rows and columns each were centered around the axial midpoint of the homogeneous CTP486. The CT‐numbers of the corresponding pixels were averaged row‐ and column‐wise, respectively, to create a mean CT‐number profile for both the AP and lateral image direction. Emerging non‐uniformities were classified as the maximum of the absolute CT‐number differences between both profile edges (defined to be at 5% and 95% profile length) and the profile center. This methodology particularly accounts for potential asymmetric CT‐number profiles. In addition to the isocentric placement of the CatPhan within the scanner, the phantom was also incrementally displaced along the gantry's rotational axis in 2 cm steps. This served to evaluate uniformity as function of the longitudinal distance from the reconstruction center.

#### 2.4.7 Dose assessment

Dose measurements were performed using the IEC 60601‐2‐44^25^ compliant standard body dosimetry phantom. The phantom has a diameter of 32 cm, is made of acrylic glass, and was placed isocentrically within the scanner on a carbon fiber table. It features one central and four peripheral drill holes, into which a 10 cm pencil ionization chamber of type 30009 (PTW‐Freiburg, Freiburg, Germany) was sequentially inserted for measuring the corresponding dose length products (DLPs). The manufacturer calibrated the detector with a reference radiation quality of 120 kV and 8.4 mm aluminum half‐value layer and specified its energy response to ≤5% for tube voltages of 70–150 kV. Unused drill holes of the phantom were filled with acrylic rods.

Based on $\mathrm{DL}P_{\mathrm{central}}$ measured in the central drill hole and $\bar{\mathrm{DL}P_{\mathrm{peripher}}}$ as average of the DLPs obtained in the four peripheral holes, the weighted cone‐beam dose index (CBDI_w_)^26^, ^27^ indicating the mean point dose measured over the chamber length was calculated:

(4) $$\mathrm{CBD}I_{w}=\frac{\mathrm{DL}P_{\mathrm{central}}}{3\;\cdot 10\;\mathrm{cm}}+\frac{\;2\;\cdot \bar{\mathrm{DL}P_{\mathrm{peripher}}}}{3\;\cdot 10\;\mathrm{cm}}.$$

The CBDI_w_ was measured three times for each investigated scan protocol.

### 2.5 Anthropomorphic phantom studies

In addition to the evaluation of physical parameters, the imaging performance of the IRm was also assessed using an anthropomorphic upper torso Alderson® phantom (dimensions: AP = 24 cm, lateral = 31 cm, cranial‐caudal = 72 cm; Radiology Support Devices, Long Beach, CA, USA). The phantom comprises, embedded into soft tissue simulating Rando‐plastic, an artificial adult skeleton as well as integrated intestine, bladder, and lung structures and imitates a human thorax, abdomen, and pelvis.

Furthermore, 15 flexible plastic catheters (Type 6F; Elekta, Veenendaal, Netherlands) were implanted into an anthropomorphic breast phantom (Breast Elastography Phantom Model 059; CIRS, Norfolk, VA, USA) by using brachytherapeutic guidance needles. The catheters were fixed to the breast's skin with appropriate plastic buttons to prevent slippage. The prepared breast was placed on the thorax of the Alderson® phantom to simulate the female anatomy during breast examinations (Figure 1).

The visual assessment of the CBCT‐scans allowed to evaluate the IRm's imaging performance in simulated clinical patient examinations. The acquired scans were interpreted with respect to the measured physical imaging parameters.

## 3 RESULTS

### 3.1 Geometric reproducibility and accuracy

**FIGURE 3:**
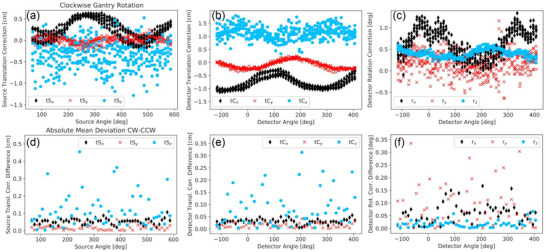
The three translational corrections of focal spot (a) and detector midpoint (b) as well as the detector rotation corrections (c) obtained for clockwise gantry rotation in all eight calibration procedures. Furthermore, the differences of the corrections obtained for clockwise and counter‐clockwise gantry rotation were calculated for each calibration and then averaged per angle over all eight measurements. The absolute values of these averages are illustrated in (d‐f), where no error bars are shown for clarity

The three focal spot and six detector corrections obtained for the clockwise gantry rotation direction in all eight calibrations are shown in Figure 3a–c. For each single calibration, the individual corrections varied along the entire angular gantry orbit as a result of mechanical inaccuracies, vibrations/oscillations, and/or gravity effects. The mean value of all corrections averaged over the entire gantry orbit and all eight calibrations was, except of for tC*_y_*, subject to systematic offsets different from zero. This offset was up to 11.5 ± 0.4 mm (tC*_z_*) for the translational corrections and up to 0.64 ± 0.02° (*r_x_*) for the rotational corrections. Similar systematic offsets were found for the counter‐clockwise rotation direction, and are therefore not shown for brevity.

As can be seen from Figure 3a–c, the calculated corrections varied considerably between the eight calibrations, even at fixed angles. For quantification, the standard deviation of the results obtained in all eight calibrations was calculated for each correction at each angle. The maximum angle‐specific standard deviations ranged from 1.0 mm (tS*_y_*) to 5.3 mm (tS*_z_*) for the translational corrections and from 0.06° (*r_z_*) to 0.61° (*r_y_*) for the rotational corrections. Averaged over the entire orbital range, the mean standard deviations ranged from 0.6 ± 0.2 mm (tS*_y_*) to 2.1 ± 0.7 mm (tC*_z_*) and 3.0 ± 0.9 mm (tS*_z_*) for the translational corrections and from 0.039 ± 0.011° (*r_z_*) to 0.23 ± 0.09° (*r_y_*) for the rotation corrections, respectively. Especially the results for tS*_z_* and *r_y_* appeared to be unreproducible as shown in Figure 3a,c. Again, similar results were obtained for counter‐clockwise gantry rotations.

**FIGURE 4:**
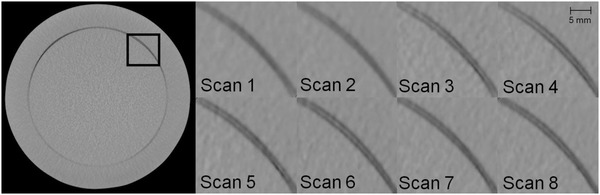
Double contours observed at the inner CTP486 edge, exemplary presented for the region marked black on the left image. The appearance of the double contours varied as illustrated between the eight considered PH scans acquired in direct succession, thus indicating geometric instabilities varying from scan‐to‐scan. Windowing—Level: ‐40 HU, Width: 500 HU

In particular, the described high fluctuations were not exclusively caused by uncertainties in the software‐sided calibration routine, but by actual geometric instabilities of the IRm. This was evident by considering eight scans of the CatPhan module CTP486 performed in direct succession with the full scan protocol PH. On the inner margin of the module, significant double structures appeared. These, however, were not stable (which could be an indication of a wrong geometric calibration only), but varied significantly between the eight scans as shown in Figure 4. For short scans, these effects were much less pronounced. This observation provides evidence for fluctuating geometric properties of the scanner and thus for existing geometric instabilities.

Furthermore, for each calibration procedure and angle, the difference between the corrections obtained for clockwise and counter‐clockwise rotation direction was calculated and then averaged over all eight measurements. The absolute values of these mean deviations are shown in Figure 3d–f. Specifically for tS*_z_*, tC*_z_* as well as *r_y_*, highly fluctuating deviations between the two rotation directions were found. The deviations were up to 4.6 mm (tS*_z_*), 3.1 mm (tC*_z_*), and 0.3° (*r_y_*).

The observations that instabilities occurred particularly with the full scan protocol PH were confirmed by the examinations of imaging fidelity. For each protocol, the respective measurement results are provided in Table 3. Only minor differences were found between all short scan protocols. For these, the strongest geometric inaccuracy was measured for PS and amounted to 0.21 ± 0.18 mm. However, for the full scan protocol PH a significantly increased inaccuracy and especially measurement uncertainty of 0.39 ± 0.53 mm was obtained. This magnitude was roughly consistent with the appearance of the double structures shown in Figure 4. PH thus showed a worse imaging fidelity compared to all short scan protocols, for which altogether a reasonable geometric accuracy was obtained.

**TABLE 3:** As overview of the imaging performance of the IRm, the weighted cone‐beam dose index (CBDI_w_), contrast‐noise‐ratio (CNR) (for the LDPE, polystyrene, and Delrin® insert), non‐uniformities (determined from the lateral/anterior–posterior (AP) CTP486 computed tomography (CT)‐number profiles), image noise (from integrating the noise power spectrum (NPS)) converted to a CBDI_w_ of 1 mGy, as well as the imaging fidelity, that were altogether measured for each investigated scan protocol, are listed

		CNR			Non‐uniformity (HU)			
Protocol	CBDI_w_ (mGy)	LDPE	Polystyrene	Delrin®	Lateral	AP	Noise (HU) at 1 mGy	Imaging fidelity (mm)
Pelvis								
‐ Low dose (PL)	9.7 ± 0.3	10.4 ± 0.2	8.4 ± 0.3	14.8 ± 0.5	‐6 ± 11	‐6 ± 14	43.6 ± 0.6	0.18 ± 0.11
‐ Standard (PS)	13.6 ± 0.2	15.4 ± 0.2	10.2 ± 0.3	21.2 ± 0.8	2 ± 11	‐34 ± 10	39.9 ± 0.7	0.21 ± 0.18
‐ Obese (PO)	16.8 ± 0.2	17.0 ± 0.4	10.3 ± 0.3	20.8 ± 0.7	‐20 ± 10	‐35 ± 7	41.3 ± 0.8	0.19 ± 0.16
‐ High‐quality (PH)	16.8 ± 0.2	21.5 ± 1.6	14.7 ± 1.7	28.1 ± 2.9	‐2 ± 15	‐3 ± 23	35.2 ± 1.3	0.39 ± 0.53
Breast								
‐ Standard (BS)	5.0 ± 0.2	7.5 ± 0.5	5.6 ± 0.2	10.5 ± 0.5	‐2 ± 12	‐2 ± 17	43.8 ± 0.9	0.19 ± 0.23
‐ Obese (BO)	7.8 ± 0.3	10.4 ± 0.3	7.2 ± 0.3	14.1 ± 0.5	9 ± 10	12 ± 11	39.7 ± 0.9	0.17 ± 0.12
‐ High‐quality (BH)	8.7 ± 0.2	10.9 ± 0.2	8.3 ± 0.2	14.8 ± 0.4	1 ± 10	3 ± 18	39.7 ± 1.4	0.20 ± 0.15
Abdomen								
‐ Low dose (AL)	2.0 ± 0.2	1.79 ± 0.04	1.41 ± 0.04	2.39 ± 0.05	120 ± 60	136 ± 19	115.8 ± 0.9	0.18 ± 0.14
‐ Standard (AS)	11.9 ± 0.4	4.23 ± 0.09	3.22 ± 0.06	5.67 ± 0.10	144 ± 16	131 ± 6	113.6 ± 0.2	0.18 ± 0.14
‐ Obese (AO)	40.6 ± 0.5	8.59 ± 0.09	6.25 ± 0.06	11.44 ± 0.15	‐45 ± 10	‐40 ± 5	122.9 ± 1.3	0.19 ± 0.18

Abbreviations: AP, anterior–posterior; HU, Hounsfield Unit.

### 3.2 Dose measurements

The results of the dose measurements are summarized in Table 3. The CBDI_w_ ranged from 5.0 to 8.7 mGy and 9.7 to 16.8 mGy for the breast and pelvis protocols, respectively, and from 2.0 to 40.6 mGy for the abdomen protocols. Except of for PO, PH, and AO, the measured doses were below the diagnostic reference values^28^ for CT thorax or abdomen–pelvis examinations. However, it has to be noted that radiation exposure should always be interpreted with respect to clinical requirements and therefore higher dose levels (e.g., for obese patients or required high CNR) may be justified as well. In each case, the measured CBDI_w_ was consistent with the radiation exposure of comparable CBCT‐systems^6^, ^29^ and, with the exception of the AO protocol, also conventional multi‐slice CT‐systems.^30^ The ratio between the DLPs measured in the peripheral and central phantom holes was for the abdomen protocols increased by a factor of up to 2.3 compared to the breast and pelvis protocols. This indicated that the air prefiltering selected by the manufacturer increased the patient surface exposure with significantly less primary radiation contributing to the actual imaging process.

### 3.3 Image uniformity and CT‐number accuracy

**FIGURE 5:**
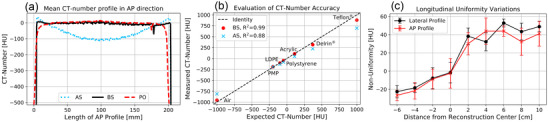
The anterior–posterior (AP) computed tomography (CT)‐number profiles obtained for the AS, BS, and PO protocols (a). The AS protocol showed significant cupping, whereas the phantom edges were blurred for the PO protocol. Furthermore, the CT‐numbers measured for the CTP404 inserts are plotted exemplarily for BS and AS (b), where for BS a high compliance (*R* ^2^ = 0.99) with the expectations was achieved. Furthermore, non‐uniformities (determined for both the lateral and AP image direction) depended distinctly on the distance to the longitudinal zero position (c), revealing significant variations within the scan range

As shown in Table 3, the non‐uniformities measured in both lateral and AP image direction averaged <12 Hounsfield Unit (HU) for almost all in‐house protocols. Thus, the IRm showed a very high image uniformity in the central axial beam plane in these cases. Exceptions were the PS and PO protocols. For these, negative non‐uniformities (i.e., higher CT‐numbers in the center of the calculated profile than at its edges) of up to ‐35 HU were observed. These resulted from the comparatively high radiation exposure reaching the detector, which led at the edges of the CatPhan to detector oversaturation. Insufficiently corrected in image reconstruction, this resulted in a slight smearing and corresponding CT‐number reduction of the phantom margins explaining the measured non‐uniformities. For the PS protocol, this effect was observed only in AP direction due to the 2.5:1 velocity modulation in the scan trajectory. In more central phantom regions, a high image uniformity comparable to the other in‐house protocols was obtained also for PS and PO. For visualization, the AP profiles calculated for the BS and PO protocol are shown in Figure 5a.

While no substantial non‐uniformities were observed for any of the in‐house protocols, significant cupping of up to 144 HU was obtained for the manufacturer's abdomen protocols AL and AS (see Table 3), which was founded in inadequately adjusted scatter corrections. It has to be noted, that the manufacturer uses, to our knowledge, only heuristic object scatter methods. For illustration, the AP profile of the AS protocol is also shown in Figure 5a. For the AO protocol, however, a high uniformity roughly consistent with the in‐house protocols was measured in the more central phantom regions, but the detector saturation issue described above occurred at the CatPhan edges.

In addition to the high uniformity, the in‐house protocols as well as the AO protocol revealed a very good CT‐number accuracy. The CT‐numbers measured for the CTP404 inserts are plotted above the expectations in Figure 5b, exemplarily for the BS protocol. Very similar results were obtained for the other in‐house protocols and AO, and are not shown for brevity. In each of these cases, a good agreement (coefficient of determination *R* ^2^ > 0.97) between measurements and expectations and high CT‐number linearity were observed. The CT‐numbers measured using the AL and AS protocols deviated stronger from the expectations (*R* ^2^ of 0.90 and 0.88, respectively) as exemplarily shown for AS in Figure 5b.

Furthermore, non‐uniformities significantly varying along the gantry's rotational axis were observed when shifting the phantom incrementally along that axis. The non‐uniformities calculated for the BS scans at each phantom position for both the lateral and AP image direction are shown in Figure 5c. Within the entire scan range, uniformity variations of up to 75 ± 23 HU became evident. Along its longitudinal scan range, the IRm thus did not show high image uniformity.

### 3.4 Noise and CNR

**FIGURE 6:**
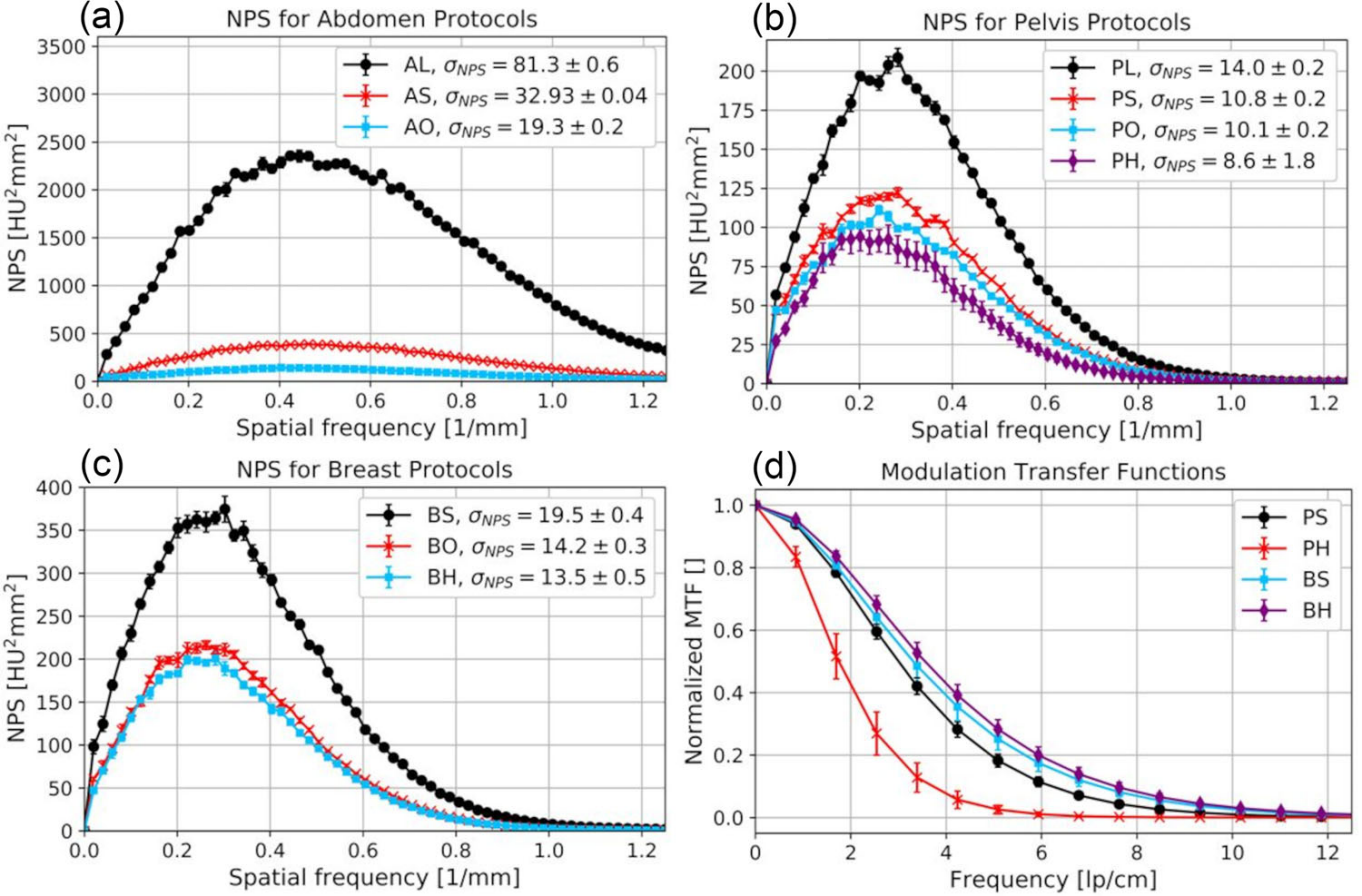
(a–c) The 1D noise power spectrum (NPS) calculated for all protocols. The overall noise obtained by appropriate integration is indicated in each case. For the manufacturer protocols, a significant amplification of the noise was found, particularly also at higher frequencies. The calculated modulation transfer functions (MTFs) are shown exemplarily for the PS, PH, BS, and BH protocols in (d). The rapid drop in the MTF particularly of the PH protocol demonstrated the significant impact of the geometric instabilities of the IRm on spatial edge resolution. Note, that the resolution is mainly determined by the sampling pixel size of the initial image reconstruction

The 1D NPS calculated for each scan protocol is shown in Figure 6a–c, with the overall image noise obtained by corresponding integration being specified in each case. For the breast and pelvis protocols, noise with comparatively lower frequencies of essentially less than 0.8/mm occurred. With increasing radiation exposure, the NPS fell flatter towards high frequencies, indicating a reduction particularly of the lower frequency noise. The measured total noise was <19.5 ± 0.4 HU and <14.0 ± 0.2 HU for all breast and pelvis protocols, respectively, with the lowest noise of 8.6 ± 1.8 HU obtained for the full scan protocol PH.

The manufacturer's abdomen protocols showed the highest image noise in the performed examinations, partly also based on the selected tube voltage of only 100 kV. The maximum noise was found for AL and amounted to 81.3 ± 0.6 HU. The use of the RamLak kernel with cutoff 1.0 resulted in increased higher frequency noise reaching up to the Nyquist frequency, thus leading to aliasing artifacts.

As becomes evident from the image noise converted to a CBDI_w_ of 1 mGy shown in Table 3, the prefilter‐choice had a significant impact on the results. For the air filtering of the abdomen protocols, the highest converted noise of up to 123 HU was measured. This was about three times higher than the results of the in‐house protocols. Taking the pelvis protocols as example, starting from 0.2 mm Cu filtration of the PL (particularly taking the use of the smoother Cosine kernel into account), over 0.5 mm Cu of the PS and PO, up to 1.5 mm Cu of the PH protocol, a significant decrease of noise from about 44 to 35 HU was observed. Similar observations were made for the breast protocols, where the usage of different kernels and tube voltages has to be considered additionally. In particular, it has to be noted that using the full scan protocol PH a reduction of converted noise compared to the short scan protocol BH acquired with the same kernel and prefilter was achieved.

The CNRs obtained for the LDPE, polystyrene, and Delrin® insert, shown in Table 3 for each protocol, showed a continuous enhancement with rising radiation exposure. This CNR behavior was compliant to the aforementioned noise results. In particular, for fat (LDPE) and bone (Delrin®), high CNRs of up to 21.5 and 28.1 were measured, respectively. No significant differences in the individual insert‐background‐contrast between the investigated protocols was found.

### 3.5 Spatial resolution

The geometric instabilities described above had a strong impact on the spatial resolution of scans. For instance, for the full scan protocol PH, as shown in Figure 6d, the most steeply decreasing MTF and thus worst spatial resolution was obtained. In this case, the limiting spatial resolution *f*_lim_ (defined as frequency at which MTF(*f_x_*) drops to 10%) was only 3.8 lp/cm. This observation was consistent with the abovementioned misalignment artifacts, which were particularly pronounced for full scans. The blurring and loss of the edge contrast resulted in strong reductions of the spatial edge resolution.

Albeit much less pronounced than for full scans, the impact of geometric instabilities on spatial resolution was also identifiable for short scans. This became evident as, despite variations in the reconstruction kernels between the PS, PO, BO, AL, AS, and AO protocols, no significant differences between the respective MTFs were found. Exemplarily, the MTF calculated for the PS protocol is also shown in Figure 6d. This observation indicated that the spatial resolution of the IRm was strongly influenced and, in particular, significantly limited by the described geometric instabilities.

The MTFs calculated for the BH, BS, and PL protocols (shown in Figure 6d are the curves for BS and BH) differed not substantially from each other, but from the abovementioned results. For the BH protocol, this was assumed to be a result of the improved angular sampling. For the BS and PL (and the AL) protocols, the IRm used its small focal spot resulting in improved resolution. However, no such resolution improvement was observed for the AL protocol, presumably due to the very high image noise (see Figure 6a) occurring at the examined edge in this case.

### 3.6 Anthropomorphic phantom scans

**FIGURE 7:**
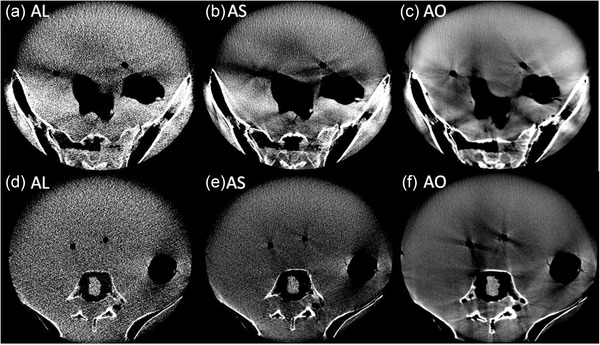
Two representative axial slices (bottom and top rows, respectively) acquired with the AL (a and d), AS (b and e), and AO (c and f) protocols. The AL and AS protocols were particularly characterized by high image noise. On all scans, significant artifacts occurred at the transitions between different phantom structures. Windowing—Level: 60 HU, Width: 400 HU

The scans of the anthropomorphic Alderson® phantom acquired with the manufacturer's abdomen protocols are shown in Figure 7 for two different, representative axial slices. On all scans, distinct differentiations and delineations of bones from the surrounding tissue were possible. However, as can be seen from Figure 7a–c/f, distinct scatter and hardening artifacts originated from bone/air–tissue transitions (e.g., specifically at intestinal gas bubbles), and considerably impaired the CT‐number accuracy and image uniformity. The artifacts were most pronounced for the AO protocol, which showed the least noise of the abdomen scans. For the AL and AS protocol, the artifacts were partially masked by the high image noise. Misalignment artifacts in the form of streak and shadow artifacts became evident.

**FIGURE 8:**
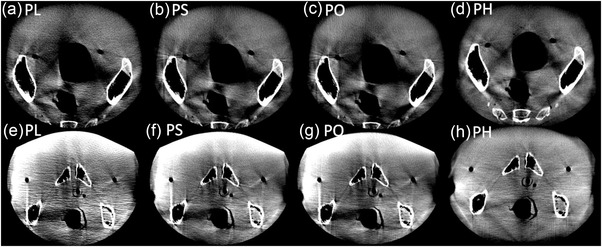
Axial slices of the upper and lower pelvis (top and bottom rows, respectively), acquired with the pelvis protocols PL (a and e), PS (b and f), PO (c and g), and PH (d and h). Again, significant artifacts appeared at structural interfaces which, however, could be reduced with increasing prefilter strength. The PH protocol also showed comparatively more pronounced blurring and double structures. Windowing—Level: 60 HU, Width: 400 HU

Comparable hardening and scatter artifacts as well as CT‐number inhomogeneities also appeared for the pelvis protocols. Figure 8 shows representative axial slices of the lower and upper pelvis, respectively. Particularly in the upper pelvis, high density differences between bone, tissue, and gas‐filled rectum/bladder, as well as the high absorption of the pelvic bones caused severe artifacts. These were slightly reduced by using the full scan protocol PH with its 1.5 mm Cu prefiltering and extended scan time. The usage of stronger prefilters resulted, especially for the lower pelvis, in significant image quality improvements. However, in this anatomical region the skin of the Alderson® phantom, that is, the interface between air and tissue, showed substantial artificial CT‐number increases. For PH, the increased geometric instabilities resulted in enhanced blurring at tissue transitions.

**FIGURE 9:**
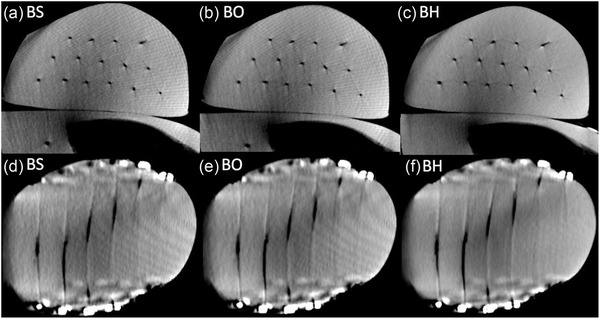
Scans of the CIRS breast phantom acquired with the BS (a and d), BO (b and e), and BH (c and f) protocols in axial (top row) and coronal (bottom row) views. Significant artifact reduction was achieved with the BH protocol. However, all scans fulfilled the clinical requirements of catheter reconstruction in the treatment planning system. Windowing—Level: ‐40 HU, Width: 900 HU

The scans of the CIRS breast phantom placed on the Alderson®’s thorax, that were acquired with each breast protocol, are shown in Figure 9. All scans showed sufficient image quality for identifying catheter paths and reconstructing catheters in the treatment planning system. The positions of each catheter could be determined accurately. Despite the occurrence of slight cupping, image uniformity within the breast was acceptable. The breast scans therefore showed adequate image quality for clinical requirements.

For all protocols investigated in this work, except of for BH and PH, undersampling artifacts occurred on the scans. These were due to the low detector's frame rate of only 12 Hz, coupled with the acquisition times of BS and BO of only 17 s and the 2.5:1 velocity modulation (here the undersampling artifacts were pronounced in the AP direction) used for the abdomen and most of the pelvis protocols.

## 4 DISCUSSION

The technical assessment of the IRm revealed significant geometric instabilities of the device. With the exception of tS*_y_*, significant offsets were measured for all corrections, which were up to a factor of 40 higher than for the table‐mounted ImagingRing system also built by medPhoton.^21^ This may be seen as direct result of the mobile device configuration. However, as already described by Keuschnigg et al.,^21^ the measured offsets prove a mismatch between the nominal geometrical device model and the real geometric properties of the IRm, for example, with respect to source‐detector‐distance deviations (tS*_z_*, tC*_z_*), non‐exact assignments of actual source/detector‐positions to acquired projections (tS*_x_*, tC*_x_*) or mechanical detector‐tilt (*r_x_*). The exact meaning of the individual offsets is not discussed here for brevity, but is described in detail by Keuschnigg et al.^21^ As also reported by this group, the calibration performance for both rotation directions separately is considered important, for example, to account for potential elasticity/strength hysteresis effects of the scanner component mounts.

The observed offsets indicated the necessity of performing a geometric calibration of the IRm to achieve a match between the device's nominal and actual imaging geometry. This forms the basis for accurate image reconstructions.^19^, ^20^ However, our investigations showed only a low reproducibility of the calibration procedure, with fluctuations of the obtained corrections of up to several millimeters between individual measurements. The fluctuations were generally more pronounced in the *z*‐direction than in the *x*‐ and *y*‐directions. As described by Keuschnigg et al.^21^, this is likely caused by the arrangement of the ball bearings on the cylindrical phantom surface. Due to this, the exact marker detection is geometrically constrained associated with stronger uncertainties along the projection direction than in the planar projection plane. Anyway, it has to be noted that the sampling distance during the calibration routine was about 180 μm in the gantry center (the 2 × 2 detector binning is used standardized) and thus the observed correction fluctuations were significant and not negligible. The measured variations were up to more than a factor 10 larger than for the table‐mounted ImagingRing system.^21^ Also compared to mobile C‐arm systems, the corrections showed partly significantly higher variations as well as a lower reproducibility.^6^

The double structures observed at the inner CTP486 margin, varying considerably between the individual scans, served as evidence for mechanically constrained geometric instabilities. These were more pronounced for full scans than for short scans, due to the requirement of full scan trajectories of an exact match between opposing X‐rays. In line with this, a markedly lower imaging fidelity was obtained for the full scan protocol PH than for short scans, for which altogether a reasonable geometric accuracy was measured. The reasons for these instabilities were not determined within the scope of this work. However, they are suspected in particular in oscillations of the detector due to its long portion hanging freely in air (Figure 1a) and in a variant wobbling of the scanner during acquisitions. The latter could particularly be founded in no distinctly recognizable counterweight being mounted to the source‐/detector‐opposite gantry side. This could, due to different weight distribution of these components during a scan trajectory, lead to small oscillations amplified by the gantry's large bore diameter. Other reasons for instabilities could also be mounting errors, mismatched device covers causing frictions, or a too soft clinic floor (we have a standard polyvinyl chloride (PVC) floor) giving way to the scanner's weight. A potential mismatch of gears in the gantry cable pull drive could also contribute to the instabilities. Note that instabilities related to the hardware structure may not be readily resolved by software‐sided corrections. Note again that the geometric calibration was performed using the IRm's default calibration procedure with no customer‐sided adaptions/adjustments being possible. However, based on valuable feedback from one of the anonymous referees, we think that possibilities for such adaptations, in particular taking the usage of scan protocols with different scan times and motor control (e.g., velocity modulation) into account, may be beneficial for improving the geometric reproducibility and should therefore be provided by the manufacturer.

A more detailed inspection of the instabilities causing effects has to form the scope of further investigations. This is particularly imperative, since resulting misalignment artifacts and blurring may lead in clinical patient examinations to low‐contrast transitions being non‐differentiable sufficiently, due to a lack of contrast. A significant impact on the image quality parameters as, for example, spatial resolution and imaging fidelity, was shown already in this work. Note that, especially for short scans, a reasonable reflection of geometric distances between the examined objects was achieved. The mean geometric inaccuracies were below one voxel size of the scans. Nevertheless, as reported, streak artifacts or blurring, especially at object edges, impaired the image quality. It is therefore considered important that scan parameters are adapted to these underlying constraints. For example, scans with a very high spatial resolution appear to be technically impractical at present. The implementation of higher detector binning might lead to respective reductions in the impact on image quality.

Using the in‐house protocols, the CatPhan studies showed in general a good image quality. A very high CT‐number accuracy as well as for CBCT‐systems exceptional high uniformity in the central beam plane was achieved. Uniformity problems occurred in some cases only at the phantom edges due to an inadequate detector saturation handling. However, significant uniformity variations were measured along the gantry's rotational axis. Based on their course (Figure 5), the deviations were assumed to be a result of the (spectral) anode Heel‐effect not considered in image reconstruction and varying beam hardening for non‐orthogonal X‐ray beam entry into the phantom, both leading to changing scatter conditions. Slightly different scattering conditions based solely on the different phantom positions can, in our opinion, not explain the strong variations in the results. The obtained image noise was even taking the respective CBDI_w_ into account very tolerable. Note that the noise is expected to be higher in patient examinations than in the performed phantom studies, already due to the patients’ comparatively larger sizes.

In comparison, the manufacturer's abdomen protocols showed a reduced image quality. Severe cupping caused by inappropriately adjusted scatter corrections appeared. Image noise was significantly increased due to the usage of only 100 kV tube voltage as well as particularly the RamLak kernel with cutoff 1.0, and exhibited frequencies in the Nyquist range causing aliasing. The use of 100 kV tube voltage even for the AO protocol intended for obese patients is in principle considered inadequate, due to the reduced penetration power of the respective X‐rays leading to increased absorption and scatter radiation. Moreover, the use of air‐prefiltering also results in an enhanced relative proportion of lower‐energy photons and thus an inefficient increase in noise, scatter radiation, and dose. As consequence, adjustments/optimizations of the manufacturer protocols are inevitable to meet our clinical needs.

The abdomen scans of the anthropomorphic phantom confirmed the above descriptions. However, for both the abdomen and pelvis scans significant scatter and hardening artifacts were observed. These became particularly apparent behind regions of very high absorption, such as pelvic bones, and at air/bone–tissue transitions. In these areas, the image quality of scans was significantly reduced. Consequently, the high image quality obtained for the pelvis protocols during CatPhan examinations could not be approved in the anthropomorphic phantom studies. Nevertheless, the permissible spatial resolution as well as the reasonable high‐contrast differentiability appeared to be sufficient for visualizing the position of – in comparison to the tissue – high‐contrast brachytherapy applicators, templates, and needles, as well as for bone imaging. Taking the described artifacts and non‐uniformities into account, a distinct differentiation of soft tissue structures such as individual organs appears feasible to only a limited extent. However, due to the lack of soft tissue structures within the Alderson® phantom, this assessment remains subject to further investigation and, ultimately, the first patient scans. The transferability of our results to patient examinations can only be assessed by the actual clinical operation of the device.

Both the CatPhan and anthropomorphic phantom studies revealed image quality problems at the phantom surface/skin for some scan settings. It appears therefore unavoidable to position the FOV of the scan completely within the phantom/patient in these cases. Hence, imaging with the full patient size being visible on the scan, for example, by using stitched imaging techniques, or the inclusion of surface regions, for example, required for the examination of keloids or superficial lymph nodes, may require further scan protocol developments. The targeted use of the IRm's various prefilters could play a key role in this context. Significant image quality improvements with increasing prefilter strength were also observed within the scope of this work. By the reduction of the relative fraction of lower‐energy photons significantly contributing to the generation of scatter radiation, a much better dose‐to‐noise ratio with non‐markable contrast differences was achieved. Moreover, the better penetration power of the hardened X‐ray spectrum serves for a homogenization of the dose distribution within the patient and a comparatively better protection of the skin, thus enabling a CBDI_w_ reduction at fixed image quality. However, to achieve a certain dose level within the patient, a higher prefilter strength requires also an increase in the initial tube output, thus leading to an increased system's heat load and consequently a lower system lifetime. For instance, the PH protocol exhausts the system's maximum heat load by up to 80% with only one single scan, and several minutes of waiting time are required to perform multiple scans in direct succession. It is therefore a matter of careful user consideration to find compromises between heat load, image quality, and radiation exposure.

The investigated breast protocols provided sufficient image quality for catheter reconstruction in the treatment planning system. All catheters were not affected by noise in their appearance and showed good contrast to the tissue. The observed stripe and undersampling artifacts affected the circularity of the catheters, but this did not further impair the identification of the catheter paths. In particular, the BH protocol provided a very good image quality with higher freedom from artifacts compared to BS and BO, due to the use of 1.5 mm Cu prefiltering as well as improved angular sampling. However, for breast examinations, it is essential to give breathing commands to the patient, to avoid catheter blurring or further motion artifacts. In this respect, holding the breath for BH's acquisition time of 30 s is considered hardly achievable, especially for elderly and multi‐morbid patients. Splitting an BH scan into two scan phases of 15 s each could be a smooth solution. However, note that, in order to avoid catheter blurring or the occurrence of duplicated catheters, the respective scans would have to be acquired in exactly the same breathing phase and breast position, which seems feasible from our CBCT experience in external beam radiation therapy using a medical linear accelerator. The evaluation of the clinical applicability of the individual protocols has to be the goal of further investigations and especially of the first patient scans.

The clinical utility of the IRm lies, in our opinion, particularly in the visualization of the in situ arrangement of high‐contrast brachytherapy applicators such as catheters, probes, and needles. In the present work, we found a good high‐contrast visualization and corresponding high CNR, tolerable noise, as well as, for short scans, reasonable spatial resolution and imaging fidelity. These factors are considered important for the precise detection of individual applicators. As shown by the good visualization of breast catheters, the IRm is in principle suitable to support this purpose. In this respect, the device seems well applicable in the context of adaptive brachytherapy, the essential prerequisite of which is the detection of potential applicator arrangement changes compared to the planned/intended applicator positions. The importance of such a control imaging during brachytherapy, for example, to ensure the correct treatment plan delivery, has already been described by several authors^31^, ^32^, ^33^, ^34^ and for different anatomical regions such as breast,^31^ cervix,^32^, ^33^ and prostate.^34^ Mobile imaging with the IRm, both within the surgical theatre and the treatment room, can thus trigger immediate measures for potential therapy adaptations (such as applicator adjustment or treatment re‐planning). Hence, the device enables the implementation of a workflow for image‐guided adaptive brachytherapy. Outside of brachytherapy, the device may also be suitable for image‐guided bone surgery, due to the achieved good contrast and differentiability of bones to/from tissue.

Despite the high potential and utility of the device, currently some image quality limitations exist, which we summarize in the following. At first, distinct hardening and scatter artifacts were observed, especially at bone/air–tissue transitions (e.g., intestinal gas bubbles). Geometric instabilities were measured particularly for full scans, and resulted here in reduced geometric accuracy and spatial resolution, blurring, as well as double structures. For short scans, a reasonable imaging fidelity was obtained, but misalignment artifacts, for example, in the form of stripes, appeared as well. As consequence of the instabilities, scans with very high resolution (e.g., by using the 1 × 1 detector binning) appear impracticable at present. Furthermore, scatter and oversaturation issues at the phantom surface/skin require the user to place the FOV completely inside of the body, especially at high exposure settings. All these factors lead to the assessment that, although the visualization of in situ applicator arrangement appears suitable as described above, the low‐contrast differentiation and precise delineation of soft tissues may be impaired. Further improvements of the IRm's imaging performance regarding brachytherapy applications are thus essential.

Our work focused on the initial physical–technical evaluation of the IRm without performing a direct 1:1 device comparison, also taking potential other device technologies and application fields as basis for this approach. However, this may be the subject of further investigations. Compared to other mobile CT‐systems such as the Airo® (Brainlab, Munich, Germany)^35^, ^36^ or the BodyTom® (Samsung, Seoul, South Korea),^37^ the IRm features an enlarged gantry clearance, battery‐powered mobility, and the ability for non‐isocentric imaging, thus increasing the flexibility and mobility of the device in comparison. In our opinion, these points provide a considerable clinical benefit of the IRm. The large gantry enables in particular (intraoperative) imaging in the lithotomy position, a procedure that was previously only limited possible with other gantry‐based CBCT/CT‐systems. This also means that CBCT‐scans can be performed directly in the patient's treatment position. The battery‐powered operation of the device allows to quickly change examination rooms. Thus, imaging can be performed both within the surgical theater during applicator insertion and within the treatment room prior to irradiation fractions. The non‐isocentric imaging together with the dynamic collimation and VDW enables a reduction of radiation exposure outside the actual anatomical ROI and thus the sparing of organs at risk. Imaging is strongly facilitated by the device's tablet‐PC‐based operation, as the user is not bound to a corresponding PC workstation, and its small footprint in the ward. All scans can also be analyzed immediately after reconstruction on the tablet‐PC with regard to potential decisions on therapy adaptions. Mobile imaging by means of the IRm consequently allows, as already described above, the implementation of a smooth workflow for image‐guided adaptive brachytherapy. Due to the high effort involved (e.g., patient transfer to distant CT‐scanners), such CBCT/CT‐imaging was previously very impractical or – specifically for intraoperative purposes – not possible at all at our institution, and to our knowledge, also at many other brachytherapy sites. Along with these possibilities, the development of automated procedures for treatment plan verification and/or immediate treatment plan adjustment is aimed at. In this context, the four system cameras allow, as mentioned in Section 2.1, in principle a real‐time tracking of medical instruments (to be implemented), which may further improve the precision of brachytherapy interventions. However, it also has to be mentioned that other mobile systems, such as the Airo®, may achieve improved imaging performance compared to the IRm by enabling not CBCT, but spiral CT‐scans.^35^ In particular they also offer a fully developed image registration capability for surgical navigation systems,^36^ which currently still has to be comprehensively implemented on the IRm.

The main limitations of this work are the investigation of standard QA and anthropomorphic phantoms only, which allows to draw only limited conclusions about the IRm's imaging performance in clinical operation and actual patient examinations. Nevertheless, our work provides a significant clinical benefit, as it allows to identify the current image quality issues and, based on that, enables both customer‐ and particularly manufacturer‐sided image quality improvements. Furthermore, the results of this work facilitate the comparison of the imaging performance of the IRm as innovative, mobile CBCT‐system with other, similar systems, and gives a representation of the current technical state of the art with respect to CBCT imaging. In particular the very high CT‐number accuracy and image uniformity obtained in the CatPhan studies using the in‐house protocols was outstanding for the IRm.

## 5 CONCLUSION

With the IRm, it was possible to achieve a high image quality in the CatPhan as well as breast phantom examinations. However, the device also showed markable geometric instabilities as well as significant weaknesses in the imaging of the abdomen–pelvis region of the Alderson® phantom. Further improvements of the IRm's imaging performance are imperative.

## CONFLICT OF INTEREST

The authors declare no conflict of interest.

## AUTHOR CONTRIBUTIONS

All authors contributed significantly to the performed work and approved to final version of the manuscript to be published.

## Supporting information

Supporting Information

Supporting Information

Supporting Information

Supporting Information

## Data Availability

The data that support the findings of this work are available from the corresponding author upon reasonable request.
